# Spinal Cord Stimulation to Treat Unresponsive Cancer Pain: A Possible Solution in Palliative Oncological Therapy

**DOI:** 10.3390/life12040554

**Published:** 2022-04-07

**Authors:** Federica Paolini, Gianluca Ferini, Lapo Bonosi, Roberta Costanzo, Lara Brunasso, Umberto Emanuele Benigno, Massimiliano Porzio, Rosa Maria Gerardi, Giuseppe Roberto Giammalva, Giuseppe Emmanuele Umana, Francesca Graziano, Gianluca Scalia, Carmelo Lucio Sturiale, Rina Di Bonaventura, Domenico Gerardo Iacopino, Rosario Maugeri

**Affiliations:** 1 Neurosurgical Clinic, AOUP “Paolo Giaccone”, Post Graduate Residency Program in Neurologic Surgery, Department of Biomedicine Neurosciences and Advanced Diagnostics, School of Medicine, University of Palermo, 90127 Palermo, Italy; lapo.bonosi@gmail.com (L.B.); robertacostanzo3@gmail.com (R.C.); brunassolara@gmail.com (L.B.); umberto.emanuele.benigno@gmail.com (U.E.B.); massimiliano.porzio1@gmail.com (M.P.); rosamariagerardimd@gmail.com (R.M.G.); robertogiammalva@live.it (G.R.G.); gerardo.iacopino@gmail.com (D.G.I.); rosario.maugeri1977@gmail.com (R.M.); 2 Department of Radiation Oncology, REM Radioterapia srl, 95125 Catania, Italy; gianluca.ferini@grupposamed.com; 3 Department of Neurosurgery, Cannizzaro Hospital, Trauma Center, Gamma Knife Center, 95100 Catania, Italy; umana.nch@gmail.com; 4 Unit of Neurosurgery, Garibaldi Hospital, 95100 Catania, Italy; fragraziano9@gmail.com (F.G.); gianluca.scalia@outlook.it (G.S.); 5 Fondazione Policlinico Universitario A. Gemelli Istituto di Ricovero e Cura a Carattere Scientifico (I.R.C.C.S.), Università Cattolica del Sacro Cuore, 00100 Rome, Italy; cropcircle.2000@virgilio.it (C.L.S.); rina.di.bonaventura@hotmail.it (R.D.B.)

**Keywords:** spinal cord stimulation, cancer pain, oncology

## Abstract

(1) Background: Treatment of cancer-related pain is still challenging, and it can be managed by both medical and interventional therapies. Spinal Cord Stimulation (SCS) is a minimally invasive technique, and its use is rapidly increasing in the treatment of chronic pain. (2) Materials and Methods: Our study aims to perform a review of the pertinent literature about current evidences in cancer pain treatment by Spinal Cord Stimulation. Moreover, we created a database based on case reports or case series (18 studies) in the literature. We analyzed a clinical group of oncological patients affected by intractable pain undergoing SCS implantation, focusing on outcome. (3) Results: The analysis of the 18 included studies in our series has shown a reduction in painful symptoms in 48 out of 56 treated patients (87.51%); also 53 out of 56 patients (96.64%) have shown an improvement in their Quality of Life (QoL). (4) Conclusions: Spinal Cord Stimulation can be considered an efficient method in the treatment of cancer-related pain. However, literature regarding SCS for the treatment of cancer-related pain is largely represented by case reports and small case series, with no effective population studies or Randomized Controlled Trials demonstrating the efficacy and the level of evidence. Further prospective studies are needed.

## 1. Introduction

Spinal cord stimulation (SCS) for the treatment of chronic pain is rapidly evolving together with technological improvement and pathophysiological insights, and recent studies are proving the clinical effectiveness of SCS in other conditions as the management of cancer-associated pain.

Over half of all cancer patients will experience severe, unmanageable pain during their disease, and its treatment is a primary challenge because it is related to poor physical outcome. A meta-analysis of several studies concluded that 38.0% of all cancer patients reported moderate to severe pain (Numerical Rating Scale score ≥5) [1].

Most of these patients suffer from moderate to severe pain; a significant percentage of those with advanced-stage cancer show an increased emotional distress, impairment of their quality of life (QoL), and disability [2].

Cancer-associated pain can be related to the primary tumor, typically somatic pain in nature, but it can derive also from metastases, treatments, and diagnostic procedures. Moreover, even after cancer survival, between 20% and 50% of patients continue to experience pain and functional limitations in the years following treatment [3].

This study aims to review the current literature about recent evidence in Spinal Cord Stimulation as cancer pain treatment.

## 2. Materials and Methods

### 2.1. Study Selection

**Figure 1:**
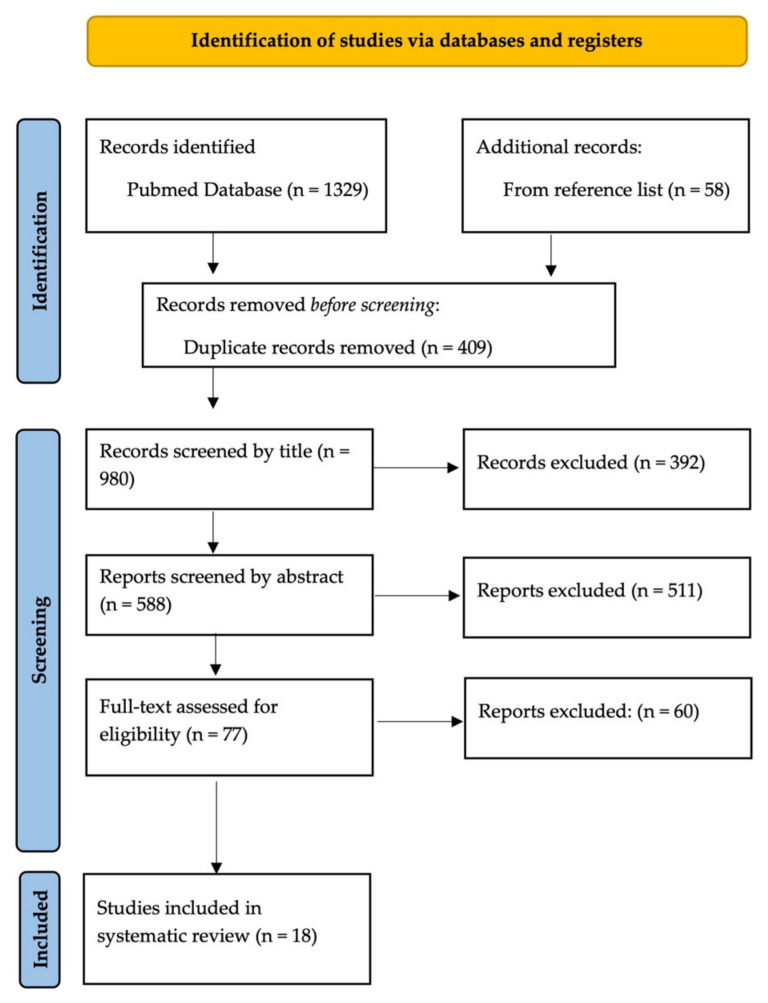
PRISMA Flow Chart.

The authors performed a systematic review of the effectiveness of Spinal Cord Stimulation in Oncologic pain treatment, following the PRISMA guidelines (Preferred Reporting Items for Systematic reviews and Meta-Analysis) (Figure 1).

An accurate search to identify pertinent articles was performed using PubMed database. The references sections of included articles were analyzed, too.

Mesh terms used were:

- “Spinal cord stimulation AND oncology”, 404 articles.
- “Spinal cord stimulation AND cancer pain”, 252 articles.
- “Spinal cord stimulation AND tumor”, 673 articles.

In our review we included case reports, case series, cohort prospective and retrospective studies, and clinical trials which have been published between 2000 and 2021; only English articles were used. To be included, studies had to analyze a single or group of patients, focusing on Spinal Cord Stimulation in treatment of cancer-related chronic pain and its clinical relevance. We aim to investigate and highlight the current state of the art in SCS as oncological pain treatment, focusing on indications and outcome.

### 2.2. Data Extraction

We created a database based on the previously selected case reports or case series (Table 1). We analyzed a clinical group of oncological patients affected by intractable pain undergoing SCS implantation: we analyzed patients’ gender and age, study group size, study design, cancer diagnosis, pain etiology, stimulation mode, pre-operative and post-operative Visual Analogic Scale (VAS) for pain evaluation, outcome in terms of pain reduction, improvement in QoL, and reduction in drug therapy after SCS implant.

Moreover, we conducted statistical analyses to evaluate differences between pre-operative and post-operative pain.

## 3. Results

### 3.1. Systematic Review

A total of 1387 studies were identified through PubMed database and references section screening (978 articles after duplicates removal).

First, articles were selected by the presence in their title of the words “spinal cord stimulation” associated with “oncology”, “cancer”, or forms of cancer (e.g., lung cancer), “tumor”, “oncological pain”, and “cancer pain”. After screening by title, we rejected 392 articles.

In total, 586 abstracts were screened according to the selection criteria. Thus, we identified 75 studies.

Next, 60 studies were rejected due to the lack of reported data about patient outcome. Finally, we included in this systematic review 18 articles, summarized in Table 1.

### 3.2. Outcome Database

All the selected records were used to structure the database to evaluate SCS outcomes in Table 2.

Patients included totalled 56, 30 males and 26 females. The mean age was 54.21 ± 8.9 years old. Cancer diagnosis and location was extremely heterogenous in the different studies.

A total of 8.92% (5/56) patient suffered from cancer-related pain, while 91.07% (51/56) suffered from treatment-related pain (surgical, chemo, or radiotherapy).

The analysis of studies included in our series has shown a reduction in painful symptoms (≥50% reduction from pre- to post-operative VAS) in 85.71% (48/56) of treated patients, and 94.64% (53/56) showed an improvement in their QoL.

SCS was associated with a reduction in drugs intake in 35.71% of patients (20/56), and to a complete stop in 46.42% of patients (26/56).

Analyzing the mean value of pre-operative (7.63/10) and post-operative VAS (2.18/10), a significant difference in pain relief between pre- and post-SCS (*p* < 0.001) was found.

## 4. Discussion

### 4.1. Treatment of Cancer Pain

Nowadays, pain is considered “the fifth vital sign”, and it must be treated as an illness itself, using a multimodal approach. [22,23] Chronic pain management, in its several forms, is still challenging. As regards musculoskeletal chronic pain, its treatment is considered by World Health Organization as a priority, due to its prevalence, health, and economic costs [24].

Opioids are the most used drugs in the treatment of chronic pain, even if their benefits are proven only in short term therapies. Less robust benefits are proved as regard patient functional outcomes. Long term opioids use is related to low Quality of Life (QoL) and an increase in severe side effects (even overdose), and its safety has not been proven to date [25].

Cancer-related pain represents an important public health problem in terms of number of patients affected and health care costs [8]. Pain is strongly related to depression, decreased quality of life and ability to perform activities of daily living, and lower adherence to treatments [26].

Management of cancer pain must consider the close relationship between pain and QoL [27]. Pain has been proven to be a major independent predictor of survival in cancer patients [27,28,29]. Over 50% of patients complain of moderate to severe pain despite medical treatment [30]. Reasons of mismanagement of cancer pain can be related to physicians (undertreatment due to fear of side effects, no patient education, underestimation of pain, poor communication between patients and doctors, or no application of pain control guidelines) and to patients (scarce adherence to treatment, fear of side effects, lack of communication between patients and doctors, or pain considered as “normal”) [23,27,31,32].

The American Society of Clinical Oncology and the European Association for Palliative Care summarized reviews for medical management of cancer pain; moreover, the World Health Organization (WHO) provided the three-step “Ladder” for the treatment of cancer pain [33,34,35].

Due to the recent access to data on the pathophysiological mechanisms and response to pain control, the idea to structure the management of cancer-associated pain through both medical and interventional therapies has gained attention.

Treatment of cancer pain is not free from medico-legal risks. According to recent publications, the undertreatment of pain is considered a medical error [23,36]. Not following updated guidelines, prescribing inappropriate doses of opioids, and failure to use all the means to achieve pain control represent situations that carry the risk of litigation [23,36,37].

Several publications describe a significant variance in the interventional pain management strategies for cancer pain, such as neurolytic blocks, intrathecal drug delivery systems (IDDS), vertebral augmentation, neuromodulation (including spinal cord stimulation (SCS) and dorsal root ganglion stimulation (DRG-S) for neuropathic pain, and radiotherapy; to date, neither strong evidence nor guidelines exist to support them [38,39]. Nonetheless, interventional treatments have proven effective in providing pain relief, reducing the burden of symptoms, minimizing opioid intake and its side effects, and having a low complication rate [3,40]. However, both invasive and non-invasive treatments carry the risk of complications [23].

Spinal Cord Stimulation in unresponsive cancer pain is an increasingly used technique, succeeding in obtaining good outcomes in terms of pain reduction and improvement in quality of life.

### 4.2. Pathophysiology of Oncologic Pain

The symptoms experienced by oncological patients are a consequence of cellular, tissue, and systemic changes that occur during proliferation, invasion, and metastasis, causing the nociceptive component of pain. The responding immune system also has a primary role in cancer pain [41]. These changes affect the physiological functioning of the nervous system responsible for the control and processing of the various painful stimuli both at the spinal and supraspinal levels, leading to a neuropathic component of pain [1].

Causes of cancer pain are multifactorial and complex, and are likely to vary with an array of tumor-related and host-related factors and processes. Pathophysiological mechanisms underlying cancer pain are not completely clear, but it certainly includes both a nociceptive and a neuropathic component [3].

Some studies have highlighted the role of tumor microenvironmental abnormalities, which in turn contribute to cancer pain [41,42,43,44]. Cancer cells produce nerve damage, which in turn determines a malfunctioning of the system responsible for differentiating pain information from non-painful stimuli, closely related to an alteration of the balance between excitatory and inhibitory neurotransmitters [45,46,47].

According to recent studies, in animal models, the generation of hyperalgesia and allodynia seems to be related to an imbalance between glutamatergic, GABAergic, and purinergic neurotransmitters and the expression of a variety of humoral factors [48,49,50].

Glial cells seem to play a key role in the maintenance of pain in cancer patients by releasing and responding to pro-inflammatory cytokines and chemokines, which in turn create a microenvironment favorable to the maintenance of a pro-inflammatory structure and an alteration in the perception of painful sensation [51].

Mechanisms of pain control by Spinal Cord Stimulation are not completely clear. Recent studies have proven its effectiveness in modulating neurotransmitter release in the spinal cord, and a direct suppression of spinal cells hyperactivity and neuron’s conductive properties. Moreover, electrical stimulus produced by SCS is targeting both neurons and glial cells, creating depolarization of cells membrane [5,6].

### 4.3. Spinal Cord Stimulation in Oncologic Pain 

Spinal Cord Stimulation (SCS) assumes that, by delivering an electric current at a certain frequency, intensity, and latency, the physiological functioning of pathologically hypo/hyperactivated circuits can be re-established through the neuromodulator action exerted at the level of the spinal nerve fiber bundles. Variations in parameters such as frequency, latency, and intensity are linked to different neural targets and effects [52].

Most electrophysiological studies have focused on the effect of spinal stimulation on neuronal components, especially axonal ones. Electrical stimulation can even target glial cells producing depolarization and glutamate release, which depends on amplitude and frequency. In this context, Spinal Cord Stimulation could also be exploited in the treatment of cancer pain, although further studies in this regard are essential. However, the in-depth knowledge of the mechanisms of genesis of cancer pain and the modality of action of stimulation highlights the potential of this therapeutic option in cancer patients [52,53,54].

Moreover, stimulation of the spinal cord also affects cortical and subcortical structures. Some studies demonstrated that electrical stimulation influences the activity of neurons in the thalamus and somatosensory cortices, indicating that SCS regulates the pain threshold at the thalamus and parietal association level [55,56]. Some authors have suggested that spinal cord stimulation could selectively act on neuropathic but not nociceptive pain as a result of processing at the cerebral level, diencephalon, or brainstem, rather than the spinal cord [57]. In a recent study, the use of spectroscopy MRI in a cohort of 20 FBSS patients revealed an increase in GABA concentration and a decrease of glucose levels in the ipsilateral Thalamus during spinal cord stimulation [58]. Recent preclinical studies have documented an increase in serotonin and noradrenaline levels in the median pontine nuclei (dorsal raphe nucleus) and the Locus Coeruleus, respectively, during spinal stimulation, underlining the fact that the supraspinal regulation of pain sensitivity can be controlled at various levels and by various networks, modified by the stimulation itself [59].

Several publications in the last few decades demonstrate the benefit of SCS in managing chronic pain (especially in previously failed back surgery syndrome—FBSS), but only recently large Randomized Controlled Trials (RCTs) and systematic reviews were conducted with persistently scarce meta-analysis assessing its use [60,61,62,63].

Considering the more recent field of interest in alternative interventional free-of-side-effects treatment for cancer-related pain, no effective population studies or RCTs demonstrating the efficacy and the level of evidence have been conducted. Between 10% and 50% of patients with cancer-related pain do not achieve acceptable levels of pain relief with opiates alone or in combination with conventional adjuvant analgesics [1,8,30].

The literature regarding SCS for the treatment of cancer-related pain is largely represented by case reports and small case series [3,64,65]. In a large percentage of patients, pain is not related to the tumor itself, but to its treatment (see Table 1). Moreover, many patients with primary spinal tumors or metastasis may require laminectomy, decompression, and fusion, developing pain worse than or equal to their pain prior to spine surgery; many of these patients may require treatment for FBSS. Therefore, it is suggested that SCS should be considered early in the treatment algorithm for these patients with both post-surgical lumbar and cervical radicular pain [3].

The high evidence level for use of SCS in FBSS has helped to establish the potential role of SCS in treating patients with cancer-related pain. However, the effectiveness and relative safety of SCS for cancer pain has not been adequately established [66].

### 4.4. Current Clinical Applications

SCS can be commonly used in several forms of cancer-related pain. One of the first retrospective studies was conducted by Shimoji et al. in 1993, where a series of 52 consecutive oncological patients with intractable pain was analyzed, and a SCS implant resulted in a reduction in pain of at least 50% [67].

From 2002 to 2021, 11 case reports and 7 case series are reported in the literature.

These studies show how Spinal Cord Stimulation can relieve various types of cancer-related pain [4,8,12,18], even due to osseous lesions, probably the type most painful and unresponsive to conventional therapy, due to pathological fracture or cortical invasion and tissue inflammation [16,62].

Nevertheless, pain is not only related to cancer itself, but it can arise after chemotherapy, surgery, and radiation therapy [8,10,13,19,20,21].

Treatment-related chronic pain syndromes can have different causes:

Chemotherapy-induced neuropathic pain [5,17].
Raynaud’s syndrome induced by chemotherapy [6].
Neuropathic pain after surgery [9,14].
Phantom limb pain after amputation from various types of cancer [11].
Neuropathic pain after radiation therapy [15].
Transverses myelitis after radiation [7,62].

The analysis of studies included in our series (see Table 1 and Table 2) has shown a reduction in painful symptoms, defined as ≥50% reduction from pre- to post-operative VAS, in 85.71% (48/56) of treated patients, with a strong impact on VAS reduction (from 7.63/10 to 2.18/10, *p* < 0.001). A total of 94.64% (53/56) showed a significant improvement in their QoL, and 82.14% (46/56) of patients experienced a reduction in or dismission of analgesic drugs intake. 

Most patients analyzed in our review were affected by treatment-related pain (51/56). Even in this subpopulation of cancer patients, results are promising in terms of pain reduction, improvement in quality of life, and reduction in intake of opioid medications [10,13]. These clinical benefits are associated with good psychological outcomes in terms of improvement in the ability to perform daily activities, less dependency on others, and great improvement in sleep pattern [17].

In the literature, no clear relationship between responders and no responders to SCS is found [3,68]. Nonetheless, multiple predictive factors are under study [7].

SCS seems to give better results in patients with a high percentage of neuropathic components of pain, and in peripheric form against central pain [7,9,11]. Somatosensory Evoked Potentials (SSEPs) have been studied as predictors in SCS outcome [7,69]. Psychiatric disorders such as depression and anxiety seem to be linked to poor outcome after implant [21]. This is important in considering the highest prevalence of depression in cancer patients, but also the effect of SCS in improving sleep and depressive symptoms together with pain [7,14,16]. Better results are related to a shorter duration of pain [21].

Although our results arise from case reports or case series, Spinal Cord Stimulation plays a decisive role in reducing the pain arising from cancer and its surrounding pathologies. 

There is an important need for a higher level of evidence to state the efficacy of SCS in cancer pain, particularly when considering the shortcomings of the current published literature in this area (including retrospective study designs, small patient numbers, and no inclusion of neurostimulation technological advancements). However, based on the experience with SCS in other forms of pain, and on small series and case reports in cancer pain, SCS can be a useful and effective therapy in many of the challenging cancer-related neuropathic pain syndromes such as post-radiation neuropathic pain, chemotherapy-induced peripheral neuropathies, and post-surgical pain syndromes [62].

### 4.5. Complications and Limitations 

The most common complication in SCS is related to lead migration, especially in quadripolar leads, followed by infections that, sooner or later, could lead to re-interventions. CSF leak and device failure are less common complications [70]. In our series, a low rate of complication is shown (two cases of lead migration, one case of allergic rejection of battery, and one case of infection) [11,12,21].

In patients affected by cancer-related pain, their immunosuppressed condition could increase the risk of device infection and can severely compromise wound healing [62]. Nonetheless, in our series, the percentage of complications is similar to FBSS patients. As a matter of fact, a study by Sica et al. proved the feasibility of an SCS implant even in patients affected by lymphoproliferative diseases [71].

Thus, SCS represents a simple and effective procedure; the low success rate is related to the lack of straight and rigid inclusion criteria in patients’ selection, e.g., exclusion of patients with psychological disorders.

Cost/effective ratio could be considered a limitation of this procedure. From our review, several patients experience pain not only related to cancer itself, but treatment-related, too. As we stated, SCS is not free from complications, but it is a completely reversible technique that can free cancer patients from the burden of opioid side effects. Moreover, opioids often fail with neuropathic pain, and long-term opioid therapy is linked to low QoL and life expectancy. In most cases, the use of so-called “invasive” interventional pain procedures can be less invasive than an aggressive medical therapy [10,13,20,72,73]. Therefore, if we consider the longest life expectancy of patients affected by cancer, the costs and side effects of pain drugs (almost all opioids) should be taken into account.

The major critical limitation is the lack of RCTs that cannot prove the efficacy of SCS in the management of cancer-related pain [74].

## 5. Conclusions and Future Perspectives

The latest evidence evaluation about SCS in cancer-related pain suggested a level of evidence II-3-C in patients with refractory cancer pain, and level III-C on a case-by-case basis for pain related to cancer treatment (such as chemotherapy-induced peripheral neuropathy) [3]. Literature is mostly based on case reports or case series, and there is an important need for a higher level of evidence. Nevertheless, from our review and statistical analysis on patient outcome, we can state that SCS is a minimally invasive and effective method to treat several forms of pain. In the future, we look forward to realize controlled and randomized studies to increase the evidence based levels of this high efficiency technique, and in doing so allowing an increased use of it in daily practice.

## Figures and Tables

**Table 1:** Summarize of studies reported from systematic review. N.R. = Not Reported. M = Male; F = Female; Pt = Patient; Pre-op = Pre-operative; and Post-op = Post-operative.

Author, Year	Type of Study	Patients	Mean Age	Cancer Type	Pain etiology	Pain Location	Stimulation Modality	Drugs Intake	Pre-Op VAS (1 to 10)	Post-Op VAS (1 to 10)	Improvement in QoL	Follow Up (Months)
Eisenberg, 2002 [4]	Case Report	1 F	50	Foramen magnum meningioma	Cancer-related	Right upper and lower limbs	Traditional SCS single lead	Reduction	10	1.5	Yes	N.R.
Cata, 2004 [5]	Case series	2 M	55.5	Pt.1: Melanoma (elbow); Pt.2: Ewing sarcoma	Treatment-related	Bilateral lower limb	Traditional SCS dual led	Reduction	Pt1: 4.5; Pt2: 4.6	Pt1:2; Pt2: 3.6	Yes	N.R.
Ting, 2007 [6]	Case report	1 M	48	Metastatic pancreatic cancer	Treatment-related	Bilateral upper limb	Traditional SCS dual lead	N.R.	N.R.	4	No	N.R.
Hamid, 2007 [7]	Case Report	1 M	54	Lung cancer	Treatment-related	Left lower limb	Traditional SCS single lead	Reduction	10	0,5	Yes	18
Yakovlev, 2008 [8]	Case Series	1 F, 1 M	47	Pt 1: Spinal metastasis from colon carcinoma; Pt 2: Anal squamous cell carcinoma	Pt 1: Treatment-related; Pt 2: Cancer-related	Pt 1: Right lower limb; Pt 2: N.R.	Traditional SCS dual lead	Dismission	Pt1: 7; Pt 2: 8	Pt1: 1; Pt2: 1.5	Yes	12
Lee, 2009 [9]	Case Report	1 F	40	Spinal meningioma	Treatment-related	Right lower limb	Traditional SCS dual lead	Reduction	9	1	Yes	N.R.
Yakovlev, 2010 [10]	Case Series	10 M, 4 F	54	Lung cancer	Treatment-related	Chest	Traditional SCS dual lead	10 Dismission, 4 Reduction	7.42	3.07	Yes	12
Viswanathan, 2010 [11]	Case Series	3 M, 1 F	38.75	Hemangiomatosis, rhabdosarcoma, spindle cell carcinoma, chondrosarcoma	Treatment-related	Pt1: right lower limb; Pt2: left lower limb; Pt3: left lip; Pt4: left low back	Traditional SCS dual lead	N.R.	NR	NR	Yes	29
Nouri, 2011 [12]	Case Report	1 M	57	Prostate cancer	Cancer-related	Testicular Pain	Traditional SCS dual lead	Dismission	5	1	Yes	1.5
Yakovlev, 2012 [13]	Case Series	6 F, 9 M	56	Metastatic colon cancer, anal cancer, and sacrum angiosarcoma	Treatment-related	Low back pain	Traditional SCS dual lead	8 Dismission, 5 Reduction, 2 same therapy	7.06	2.66	Yes	12
Wininger, 2012 [14]	Case Report	1 F	58	Lung cancer	Treatment-related	Right chest	Traditional SCS dual lead	Dismission	8,5	1.5	Yes	24
Elahi, 2013 [15]	Case Report	1 M	59	Prostate cancer	Treatment-related	Perineal pelvic pain	Traditional SCS dual lead	Dismission	8	1.5	Yes	10
Mirpuri, 2015 [16]	Case Report	1 F	65	Hereditary Multiple Osteochondromas (HMO)	Cancer-related	Lower extremities	Traditional SCS; Two paddle leads	Reduction	7	70–80% pain relief	Yes	6
Abd-Elsayed, 2016 [17]	Case Series	1 F	39	Breast Cancer	Treatment-related	Lower extremities	Traditional SCS dual lead	Reduction	8	95% pain relief	Yes	24
Hutson, 2017 [18]	Case Report	1 F	69	Metastatic sacrum lesion from thyroid cancer	Cancer-related	Low back pain	Traditional SCS dual lead	Dismission	N.R.	Reduced	Yes	N.R.
Maeda et al., 2020 [19]	Case Report	1 M	66	Pleural Mesothelioma	Treatment-related	Left thorax	Traditional SCS dual lead	Reduction	8	4	Yes	8
Quintero-Carreño et al., 2021 [20]	Case Report	1 F	60	Squamous cell Carcinoma (right popliteal fossa)	Treatment related	Right anterior lower limb	Traditional SCS dual lead	Reduction	9	2	Yes	3
Chung et al., 2021 * [21]	Case Series	7 F	59.57	Breast Cancer	Treatment related	Pt1: right chest and hand; Pt2: right chest and axilla; Pt3: upper extremity; Pt4: left chest and hand; Pt5: right chest; Pt6: right chest and arm; Pt7: left chest and arm	Traditional SCS dual lead	2 Reduction, 2 dismission, 2 same therapy, 1 dead	8.6	4.2	5 Yes, 1 No, 1 dead	22.2

* In this study, Pt n°3 failed the trial period with only 30% pain relief and did not proceed with implantation; Pt n°5 reported > 75% pain relief in trial period, however died before implantation surgery due to her disease.

**Table 2:** Summarize patients’ characteristics.

Characteristics	N°
Total number of articles	18
Total number of patients	56 (30 Males, 26 Females)
Mean age	54.21 ± 8.9 years old
Pain etiology	5/56 cancer related; 51/56 treatment related
Mean pre-operative VAS	7.63/10
Mean post-operative VAS VAS reduction (≥50%)	2.18/10 48/56 Yes, 3/56 No, 5/56 N.R.
Drugs intake	26 Stop, 20 Reduction, 4 Same therapy, 7 N.R., 1 dead
Improvement in QoL	53/56 patients
